# A Dynamic Approach to Assessing and Predicting AKI Risk in Patients with Aortoiliac Occlusive Disease Undergoing Aorto-Bifemoral Bypass

**DOI:** 10.3390/diagnostics16091382

**Published:** 2026-05-01

**Authors:** Anca Drăgan, Adrian Ştefan Drăgan

**Affiliations:** 1 Department of Cardiovascular Anaesthesiology and Intensive Care, “Prof. Dr. C.C. Iliescu” Emergency Institute for Cardiovascular Diseases, 258 Fundeni Road, 022328 Bucharest, Romania; 2 Faculty of General Medicine, Carol Davila University of Medicine and Pharmacy, 8 Eroii Sanitari Boulevard, 050474 Bucharest, Romania; adrian-stefan.dragan2023@stud.umfcd.ro

**Keywords:** aorto-bifemoral, acute kidney injury, major vascular surgery, SIRI, cost-effective, dynamic approach

## Abstract

**Background/Objectives**: We aim to identify the postoperative acute kidney injury (AKI) risk factors and the predictors of severe AKI derived from routine perioperative elements in patients with aortoiliac occlusive disease who underwent aorto-bifemoral bypass as arterial revascularization. This involves a dynamic assessment throughout the perioperative period. **Methods**: Preoperative, intraoperative, early postoperative and day-one-after-surgery data were retrospectively reviewed in consecutive patients who underwent elective aorto-bifemoral bypass for aortoiliac occlusive disease classified as TASC II D at the “Prof. C.C. Iliescu” Emergency Institute for Cardiovascular Diseases in Bucharest, Romania, between 2017 and 2023. **Results**: Preoperative clearance of creatinine (OR 1.037, CI95%: 1.009–1.066), the duration of the surgery (OR 1.435, CI 95%: 1.100–1.873), and the change between the day-one-after-surgery and preoperative systemic inflammatory response index (DeltaSIRI_1_preop) (OR 1.080, CI 95%: 1.012–1.152) were identified as independent risk factors for postoperative AKI in patients undergoing aorto-bifemoral revascularization for aortoiliac occlusive disease. The most severe form of AKI was strongly predicted by the number of packed red blood cells transfused (AUC 0.924, *p* = 0.001), the patient’s age (AUC 0.895, *p* = 0.001), and the duration of the surgery (AUC 0.895, *p* = 0.001). Furthermore, various routine and cost-effective variables related to the preoperative period, the early postoperative period, and the first day after surgery also demonstrated significant predictive value. **Conclusions**: We conducted a dynamic perioperative assessment of AKI associated with major vascular surgery. This aims to equip clinicians with a practical and cost-effective tool for evaluating AKI, thereby facilitating a more individualized approach to the diagnosis of this complication.

## 1. Introduction

According to findings from the Epidemiology of Surgical-Induced Acute Kidney Injury (EPIS-AKI) international multicenter study, postoperative AKI affects roughly 20% of surgical patients [1]. Urologic, cardiac, and vascular surgery procedures were most commonly associated with this complication, with about one-third of patients developing persistent AKI [1]. In this context, Sfyroeras et al. recently reported an AKI incidence of 20% in patients undergoing open surgical repair for complex aortoiliac occlusive disease, which increased to 28% in those undergoing an aorto-bifemoral bypass [2]. Therefore, AKI represents a common postoperative complication following major open vascular surgeries. It is associated with increased rates of morbidity and mortality, as well as higher hospital costs [3], particularly in patients with extensive atherosclerosis, which was closely linked to AKI [4]. Additionally, evidence consistently indicates that AKI can evolve into chronic kidney disease (CKD) [5], which places a significant burden on global healthcare. This progression occurs in a graded manner, with more severe cases of AKI greatly increasing the likelihood of developing CKD, along with higher rates of morbidity, mortality, and healthcare expenses [5].

Recognizing the significance of AKI in both short- and long-term outcomes, as well as its impact on healthcare resources, it has become crucial to identify its risk factors and predict its most severe stage in patients undergoing aorto-bifemoral bypass for TASC II D aortoiliac occlusive disease, a condition marked by extensive atherosclerosis. While various costly diagnostic tools and biomarkers have been developed to assess stress, damage, and kidney function, these resources are often not widely accessible [6,7]. Therefore, it is essential to identify more affordable and routine factors that can serve as risk factors of AKI, especially its most severe form, known as AKI stage 3.

The inflammatory indices may serve as valuable tools in this context. The neutrophil-to-lymphocyte ratio (NLR) greater than 5 was independently associated with postoperative AKI in non-cardiac surgery [8]. Additionally, the systemic inflammatory response Index (SIRI) has been linked to AKI following cardiac surgery [9]. There are currently no robust studies identifying the role of inflammatory indices as risk factors and predictors of AKI specifically in patients undergoing aorto-bifemoral bypass for aorto-iliac occlusive disease. The 2024 ESC Guidelines for the management of peripheral arterial and aortic diseases emphasized the growing importance of inflammation in atherosclerotic disease [10]. However, the guideline recognized the lack of reported data specifically related to aortic disease [10].

Patients diagnosed with aortoiliac occlusive disease have many comorbidities. Performing aorto-bifemoral bypass in this setting involves aortic cross-clamping, which can lead to rapid hemodynamic changes in intraoperative hemodynamics, volume status, and temperature, as well as increased transfusion requirements, systemic inflammation, and ischemic-reperfusion events, all of which significantly impact postoperative patient outcomes. The postoperative rise in serum creatinine levels typically appears later after the initial injury, whereas dynamic markers provide real-time updates on kidney function, which are essential for timely intervention. For this reason, a dynamic AKI assessment could be particularly beneficial in aorto-bifemoral bypass, compared to static assessments. This approach enables clinicians to enhance early detection of AKI, allowing for more informed and proactive decisions, which can reduce the likelihood of progression to chronic kidney disease. Moreover, a dynamic approach provides a comprehensive profile of the patient, resulting in personalized treatment and optimal resource utilization.

The primary objective of our study was to identify the risk factors of the postoperative AKI derived from routine perioperative elements in patients undergoing aorto-bifemoral bypass as arterial revascularization for aortoiliac occlusive disease classified as TASC II D. Additionally, we sought to identify predictors of severe AKI, specifically AKI stage 3, within this specific surgical context. Our retrospective study aimed to explore the role of inflammatory indices and specific risk scores for vascular surgery, particularly the Revised Cardiac Risk Index (RCRI) and the Vascular Study Group Cardiac Risk Index (VSG-CRI), in this context. Additionally, instead of focusing on a specific perioperative moment, we sought to conduct a dynamic assessment throughout the perioperative period. This approach allowed us to identify modifiable variables that can influence outcomes. Our goal was to provide clinicians with a practical, dynamic, cost-effective tool for assessing AKI, facilitating a more personalized approach to diagnosing this complication.

## 2. Materials and Methods

### 2.1. Study Design

**Figure 1:**
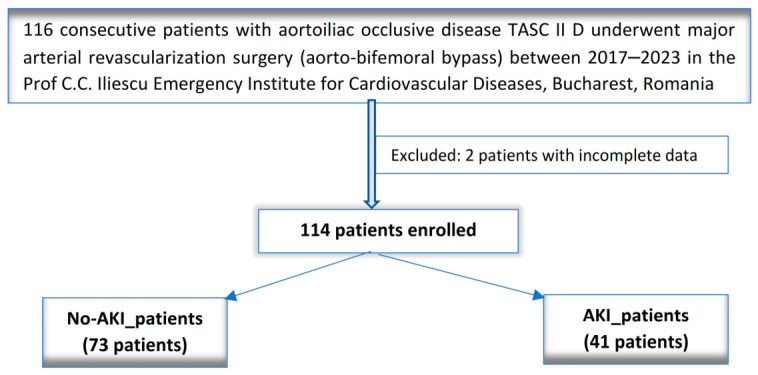
The diagram of the study.

We retrospectively analyzed 116 consecutive patients who underwent elective open major arterial revascularization through aorto-bifemoral grafting for aortoiliac occlusive disease classified as TASC II D. These surgeries were performed in the “Prof. C.C. Iliescu” Emergency Institute for Cardiovascular Diseases, Bucharest, Romania, between 2017 and 2023. Following the exclusion of 2 patients due to incomplete data, we enrolled a total of 114 consecutive patients in the analysis. The diagram of the study is presented in Figure 1. The sample size was not predetermined, as we included all consecutive patients from the designated time period. We addressed the missing data through listwise deletion. Given that only a small percentage of the data was missing (2 out of 116, or 1.72%) and was classified as Missing Completely at Random, we believe this method introduced minimal bias in our study results.

Our main objective was to identify the risk factors of postoperative AKI in patients diagnosed with classified as TASC II D (Leriche syndrome) who underwent open aorto-bifemoral bypass as revascularization surgery. Our primary endpoint was the occurrence of postoperative AKI. We analyzed two subgroups: patients who experienced AKI (AKI_patients) and those who did not (No-AKI_patients) (Figure 1). To diagnose and stage postoperative AKI, we applied the KDIGO creatinine criteria [11].

Our second objective was to identify the predictors associated with the most severe form of postoperative AKI, designated as AKI 3. The KDIGO criteria were employed to facilitate the diagnosis of AKI 3 [11]. AKI stage 3 was identified when the serum creatinine level was either three times greater than the reference serum creatinine, exceeded 4 mg/dL, or when renal replacement therapy was initiated [11].

Moreover, we explored the role of inflammatory indices and specific risk scores for vascular surgery, such as RCRI and the VSG-CRI, in this setting.

The study was conducted following the Declaration of Helsinki and approved by the Ethics and Studies Approval Committee of “Prof CC Iliescu” Emergency Institute for Cardiovascular Diseases, Bucharest (No. 14298/21 May 2024). Informed consent was obtained from all subjects involved in the study.

### 2.2. The Procedure

Patients diagnosed with aorto-iliac occlusive disease were managed perioperatively using a multidisciplinary approach by a team consisting of a vascular surgeon, a cardiologist, and an anesthetist. The preoperative evaluation included a comprehensive vascular assessment involving clinical evaluation, arteriography, and Doppler studies. Additionally, a cardiological assessment was conducted in accordance with the perioperative guidelines for non-cardiac surgery. This evaluation involved clinical assessment, EKG, echocardiography, and biological tests. The bacteriological screening and laboratory data, including hemogram, coagulation status, and evaluations of renal and hepatic function, were mandatory. Other assessments were tailored based on the patients’ comorbidities.

All the patients received the same perioperative protocol. The surgery involved an elective aorto-bifemoral prosthetic bypass with infrarenal cross-clamping of the aorta using a transperitoneal approach. Popliteal extension was performed in patients with significant multilevel occlusive disease. General anesthesia was used alongside standard and invasive intraoperative monitoring techniques. This included invasive arterial pressure monitoring with an arterial catheter, central venous pressure monitoring using a central venous catheter, and urinary output measurement. In some cases, general anesthesia was combined with a peridural approach. After surgery, patients were transported to the intensive care unit (ICU), where they were extubated after thermoregulation, hemodynamics, and respiratory function optimization and monitored accordingly.

### 2.3. Data Collection

Preoperative variables, together with postoperative data obtained at ICU admission and during the first postoperative day, were systematically analyzed. Additionally, intraoperative duration, intraoperative red blood cell transfusion data, and the performance of the popliteal by-pass extension were included in our analysis.

The hematological data related to the three studied perioperative moments (preoperatively, ICU admission and day one after surgery) consisted of the leukocytes (L), neutrophils (N), monocytes (M), lymphocyte (Lf), and platelets (P) counts, but also red cell and platelet indices: red cell distribution width coefficient of variation (RDW-CV), platelet distribution width (PDW), and mean platelet volume (MPV). Additionally, we analyzed various inflammatory indices, such as NLR, the aggregate index of systemic inflammation (AISI), the systemic inflammatory index (SII), SIRI, the platelet-to-lymphocyte ratio (PLR), and the monocyte-to-lymphocyte ratio (MLR). These indices were calculated retrospectively across the three studied perioperative moments: NLR = N/Lf; AISI = N × P × M/Lf; SII = N × P/Lf; SIRI = N × M/Lf; PLR = P/Lf, and MLR = M/Lf.

The preoperative assessment encompassed demographic information, including age and sex, the preoperative hemoglobin concentration (Hb_preop), and the identification of trophic lesions using the Leriche–Fontaine classification system. The evaluation of renal function was conducted through the calculation of preoperative creatinine clearance, utilizing the Cockcroft–Gault equation. Furthermore, hematological data, including inflammatory indices, were recorded. Patients were assessed and classified based on the VSG-CRI [12] and RCRI [13], which served as proxies for the evaluation of comorbid conditions. In the postoperative phase, hematological data and inflammatory indices were systematically collected, alongside relevant information regarding the necessity for surgical reintervention. Furthermore, the delta value for each inflammatory index was analyzed, comparing measurements obtained at ICU admission and on the first postoperative day to preoperative levels. Additionally, the ratios of mean platelet volume (MPV) to platelet count (P) and MPV to lymphocyte fraction (Lf) were computed for each of the three perioperative time points examined. We also reviewed the creatine kinase (CK) alongside the three studied perioperative moments. Postoperative data regarding changes in creatinine levels and the necessity for Continuous Renal Replacement Therapy (CRRT) were employed to evaluate the diagnosis and staging of postoperative AKI.

### 2.4. Statistics Analysis

We utilized SPSS version 31 and set a 95% threshold for statistical significance (*p* ≤ 0.05). Following an assessment of normal distribution using the Shapiro–Wilk test, we presented the quantitative variables either as mean ± standard deviation or as median and interquartile range (IQR). Subsequently, we employed either the T-test or the Mann–Whitney test to compare these variables between the two subgroups: AKI_patients and Non-AKI_patients. Categorical variables were expressed as counts and percentages (*n*%) and were analyzed using Fisher’s exact test to evaluate the occurrence of AKI. We graphically displayed the inflammatory indices (SIRI, SII, AISI, NLR, MLR, PLR) and the counts of blood cells (L, N, M, Lf, P) to illustrate their dynamics at three time points: preoperatively, early postoperatively, and the day after surgery. To evaluate the significance of the changes in these variables, we used Friedman’s test followed by the Wilcoxon signed-rank test with Bonferroni correction (*p* = 0.016). We conducted a univariable binary logistic regression analysis on each variable and assessed the significant results for multicollinearity. Variables with variance inflation factors (VIF) lower than 10 were included in the statistical model. The selected variables were then analyzed using multivariable binary logistic regression. We presented the statistical significance of the results, including the outcomes of the Hosmer and Lemeshow test. The objective of this analysis was to identify the independent risk factors for the occurrence of AKI in our cohort. We reported the odds ratio (OR), 95% confidence interval (CI 95%), and corresponding *p*-values. Additionally, we included the receiver operating characteristic (ROC) analysis of the tested model. To identify the predictors of AKI stage 3, we performed a ROC analysis that allowed us to compare the variables, including hematological data and inflammatory indices, with each other and with the risk scores relevant to the study’s objectives. We reported AUC, *p*, CI 95%, and the cut-off values derived from the Youden index, their sensitivity (Sv) and specificity (Sp), when AUC reached statistical significance.

## 3. Results

### 3.1. Data Presentation

**Figure 2:**
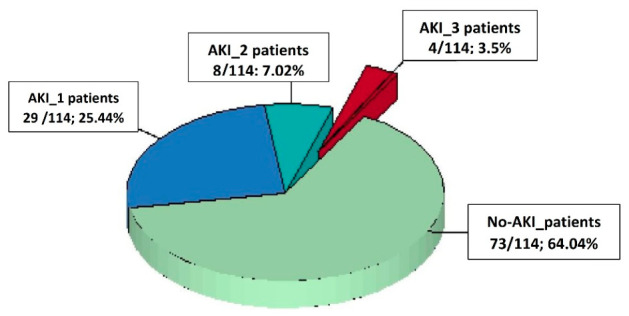
The study population distribution from the postoperative AKI point of view. Abbreviation: AKI, acute kidney injury; AKI_1–3, AKI stage 1–3; No-AKI_patients, patients without AKI.

We enrolled a total of 114 consecutive patients in our retrospective analysis (Figure 1). Postoperative AKI was diagnosed in 41 patients (41/114, 35.96%), with severe AKI (AKI 3) in 4 patients (4/114, 3.5%). All patients with AKI stage 3 died during the same hospitalization. Among the patients with AKI stage 1, there was a complete resolution of kidney function by the time of discharge. Additionally, half of the patients with AKI stage 2 also achieved complete resolution, while the other half showed only partial resolution of their AKI. The staging of postoperative AKI is illustrated in Figure 2.

The data collected in the two subgroups No-AKI_patients and AKI_ patients are presented in Table S1. Table 1 displays only the variables with statistically different values in the two subgroups, along with the clinically relevant characteristics.

Patients who experienced AKI were significantly older and exhibited significantly higher risk scores (Table 1). AKI patients presented significantly lower preoperative creatinine clearance (87 [63–102] mL/min) and underwent significantly longer surgeries (6 [4–8] h), compared to patients without AKI (101 [95.5–106] mL/min, respectively 4 [3–6] h). Platelet indices, PDW and MPV, and MPV/P showed significant differences between the two subgroups at all three perioperative time points (Table 1).

There were no notable differences between the subgroups in terms of sex, the presence of trophic lesions, rates of popliteal bypass extension, CK levels, inflammatory indices (except for NLR_1 and MLR_1), RDW, or blood cell counts (except for P_0 and P1), or the packed red blood cells transfused (Table S1).

**Figure 3:**
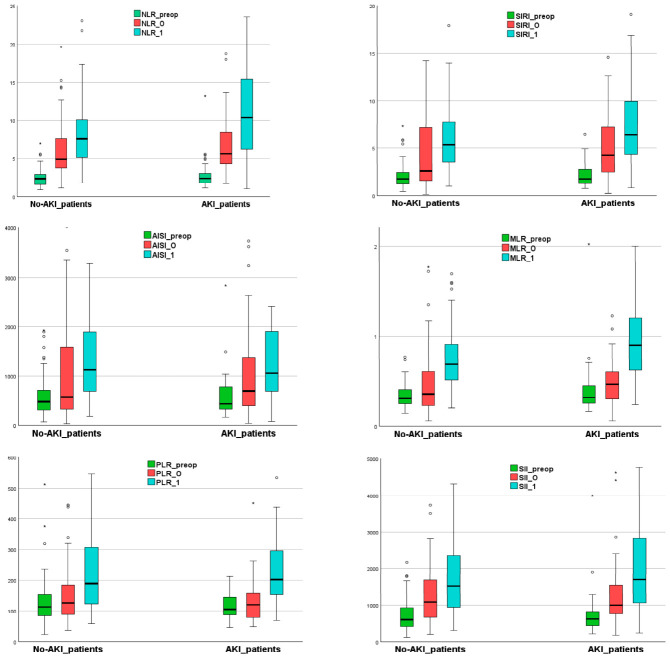
The inflammatory indices trend in patients with and without AKI (box plot representation). Note: _preop refers to preoperative variable; _0 refers to variable upon ICU admission; and _1 refers to the day-one-after-surgery variable; “◦” represents mild outliers; “*” represents extreme outliers. Abbreviation: AISI, aggregate index of systemic inflammation; AKI, acute kidney injury; MLR, monocytes to lymphocyte ratio; NLR, neutrophil-to-lymphocyte ratio; PLR, platelet to lymphocyte ratio; SII, systemic inflammatory index; and SIRI, systemic inflammatory response index.

In AKI patients, the early postoperative PLR did not differ significantly from the preoperative value (*p* = 0.141) (Figure 3).

**Figure 4:**
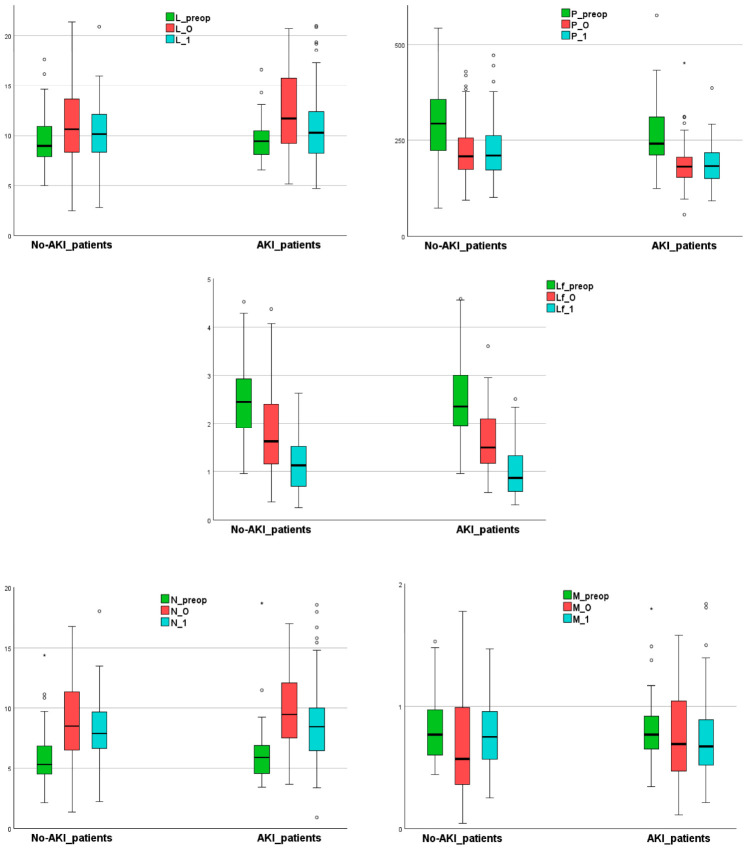
The blood cell count trends in patients with and without AKI (box plot representation). Note: _preop refers to preoperative variable; _0 refers to variable upon ICU admission; and _1 refers to day-one-after-surgery variable; “◦” represents mild outliers; “*” represents extreme outliers. Abbreviations: AKI, acute kidney injury; L, leukocytes; Lf, lymphocyte; M, monocytes; N, neutrophils; P, platelet count.

In both subgroups, Lf counts decreased significantly during the perioperative period (*p* = 0.001) (Figure 4). For P, both postoperative counts were significantly lower than the preoperative count (*p* = 0.001). The L count increased significantly from preoperative to early postoperative stages (*p* = 0.001) but then decreased significantly on the first day after surgery (*p* = 0.007). The N count showed a similar trend; however, we did not find a significant decrease on the first day after surgery compared to early postoperative levels in patients without AKI (*p* = 0.209). The M count significantly decreased from preoperative to early postoperative stages (*p* = 0.009) and then increased back to a level similar to preoperative values (*p* = 0.476) in No-AKI_patients. In contrast, the M count in the AKI group showed no significant differences during the perioperative period (*p* = 0.245).

### 3.2. Binary Logistic Regression Analysis (AKI Occurrence Endpoint)

The variables were tested in univariable binary logistic regression for the AKI-occurrence endpoint. The variables with significant results are presented in Table 2, while the entire analysis is presented in Table S2.

**Figure 5:**
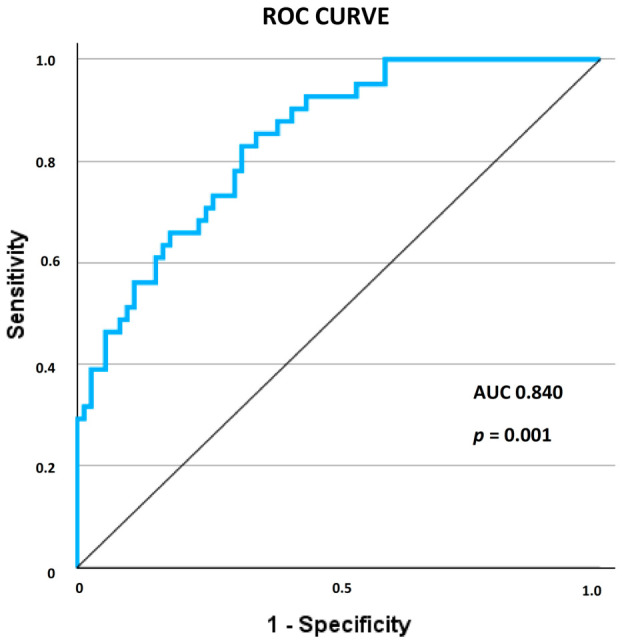
ROC curve of the multivariable regression model tested for endpoint AKI occurrence. Abbreviations; AUC, area under the curve; *p*, significance value.

Following an assessment for multicollinearity, the variables were subsequently incorporated into the multivariable analysis. The model consisted of PDW_preop, MPV_preop, PRBCs, DeltaSIRI_1_preop, Age, RCRI, VSG-CRI, Creat_clearance_preop, Intraop_time presented statistical significance (χ(9) = 41.280, *p* = 0.001), with nonsignificant Hosmer and Lemeshow test (χ(8) = 6.255, *p* = 0.619). The regression model presented a statistically significant AUC of 0.840 (*p* = 0.001, CI 95%: 0.769–0.911) (Figure 5).

The variables that exhibited significant results in multivariable analysis, and thus, emerging as independent factors associated with postoperative AKI were: preoperative clearance of creatinine (*p* = 0.009, OR 1.037, CI95%: 1.009–1.066), intraoperative time (*p* = 0.008, OR 1.435, CI 95%: 1.100–1.873), and DeltaSIRI_1_preop (*p* = 0.021, OR 1.080, CI 95%: 1.012–1.152) (Table 2). While preoperative clearance of creatinine acted as a protective independent factor, the other two factors, intraoperative time and DeltaSIRI_1_preop, were identified as independent risk factors.

### 3.3. Predictors of AKI 3

We identified that 4 out of 114 patients developed AKI stage 3. The significant results of ROC analysis are presented in Table 3, while the complete analysis can be found in Table S3.

**Figure 6:**
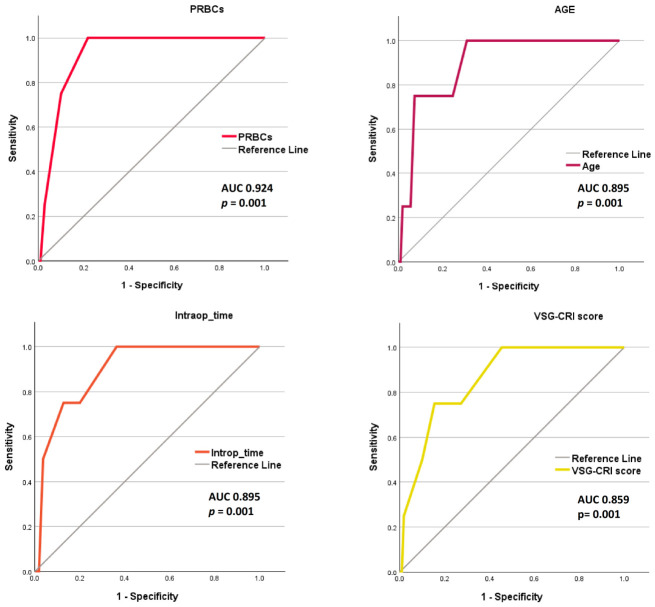
ROC curve of the variables predicting AKI 3 with an AUC of more than 0.8. Abbreviations: AUC, area under the curve; Intraop_time, surgery duration; PRBCs, packed red blood cells; VSG-CRI, Vascular Study Group Cardiac Risk Index.

The strongest predictor of AKI 3 in our cohort was the number of packed red blood cells transfused, with an AUC of 0.924. and a cutoff of 1.5 units (100% Sv, 78.2% Sp). Subsequently, the patient’s age and the duration of the surgical procedure predicted the endpoint (AUC 0.895). The age cutoff was 63.5 years (100% Sv, 69.1% Sp), while the duration cutoff was 5.5 h (100% Sv, 63.6% Sp). Figure 6 presents the ROC curve of the variables with an AUC of more than 0.8 predicting AKI3.

PRBCs, age and surgical duration outperformed the VSG-CRI (AUC = 0.859, *p* = 0.001) and RCRI (AUC = 0.741, *p* = 0.038). The VSG-CRI cutoff was 6.5 (75% Sv, 84.5% Sp) and the RCRI cutoff was 3.5 (50% Sv, 91.8% Sp). Additionally, creatinine clearance, PDW, and RDW effectively predicted AKI 3, each with an AUC of at least 0.725.

In the early postoperative period, N_0, MPV_0, RDW-CV_0, PDW_0, and L_0 effectively predicted AKI 3, with AUCs exceeding 0.725. On the first postoperative day, RDW-CV_1 and MPV/P_1 had good predictive power (0.75 < AUCs < 0.8), while PDW_1 had a weaker predictive strength (AUC < 0.7). DeltaNLR_0_preop effectively predicted AKI3 with an AUC over 0.7, while early postoperative NLR and DeltaSII_0_preop demonstrated significant results, but with lower AUC values.

## 4. Discussion

Our study revealed that intraoperative time (OR 1.435, 95% CI: 1.100–1.873) and DeltaSIRI_1_preop (OR 1.080, 95% CI: 1.012–1.152) were significant independent risk factors for postoperative AKI in patients undergoing aorto-bifemoral bypass for aortoiliac occlusive disease. Furthermore, the preoperative clearance of creatinine (OR 1.037, 95% CI: 1.009–1.066) emerged as an independent protective factor against postoperative AKI in the same surgical context. The most severe form of AKI, classified as AKI 3, was best predicted by the number of packed red blood cell units transfused (PRBCs) (AUC 0.924, *p* = 0.001), age (AUC 0.895, *p* = 0.001), and surgery duration (AUC 0.895, *p* = 0.001). Cost-effective pre and postoperative variables also showed good predictive value, underscoring the need for a dynamic assessment approach.

We conducted a retrospective study of 114 consecutive patients who underwent aorto-bifemoral bypass surgery for TASC II D aortoiliac occlusive disease. AKI was observed in 41 patients (35.95%). Among these, 4 patients (3.5%) were diagnosed with AKI stage 3. The incidence of AKI in our study was higher than the 28% reported by Sfyroeras et al. in a smaller cohort, although the rate of AKI stage 3 was similar between the two studies [2]. Additionally, Hobson et al. reported a 49% incidence of AKI in major vascular surgery, noting significant variations depending on the specific type of procedure performed [14].

We used the KDIGO definition of AKI that appears to be more effective than the definition provided by the Society for Vascular Surgery Vascular Quality Initiative [15], in the identification of AKI patients who could benefit from personalized postoperative care.

The acute kidney injury can result from various causes, including renal hypoperfusion due to ischemic-reperfusion injury, which may stem from hypovolemia, hypotension, blood loss, or embolization due to aortic cross-clamping [16,17,18]. Other contributing factors include an inflammatory response, endothelial dysfunction, nephrotoxic injury, and preoperative comorbidities such as renal artery stenosis, preoperative renal function, diabetes, and hypertension [16,17].

The acute kidney injury and atherosclerosis are interrelated conditions wherein AKI contributes to the progression of vascular disease through mechanisms such as systemic inflammation and renal dysfunction [4]. Conversely, atherosclerotic diseases, including renal artery stenosis, may precipitate the onset of AKI.

Infrarenal clamping is typically considered safer than suprarenal clamping; nevertheless, both procedures present risks for AKI attributable to factors such as renal ischemia–reperfusion injury, hemodynamic fluctuations, and systemic inflammation [19].

The preoperative renal status alongside intraoperative surgical duration are already established risk factors in the postoperative AKI setting. The EPIS-AKI study has similarly noted CKD and the duration of surgery as contributing factors to postoperative AKI [1]. According to the 20th International Consensus Conference of the Acute Dialysis Quality Initiative (ADQI), the most significant risk factor for AKI is preexisting CKD [20]. De Guglielmo et al. found that preoperative serum creatinine levels were among the independent factors that influenced the AKI occurrence in non-cardiac surgery [21]. Additionally, Chen et al. integrated surgical duration and comorbidities, including CKD, into a machine learning model aimed at predicting AKI in non-cardiac surgical procedures [22]. Furthermore, Tahir et al. recently reported that AKI incidence was significantly higher in surgeries lasting over two hours, with rates of 72.7% compared to 28.2% for shorter procedures [23]. Prolonged surgeries may lead to increased hypoperfusion, fluid shifts, and inflammatory responses.

Regarding the inflammatory indices as AKI risk factors, studies have reported SIRI in the AKI setting, but only as a single perioperative measurement rather than as a variation over time. Li et al. recently reported that SIRI served as an independent risk factor for both AKI and in-hospital mortality in critically ill patients suffering from Acute Myocardial Infarction [24]. Furthermore, SIRI has been identified as an AKI predictor in cardiac surgery, with studies showing a dose–response relationship between SIRI levels and AKI severity [9]. Chen et al. reported in their multivariate analysis that SIRI more than 1.39 was an independent predictor of contrast-associated AKI in patients undergoing elective percutaneous coronary intervention [25].

We have determined that the value of SIRI in the context of severe AKI was best interpreted not as a static measurement, but as a dynamic change observed from the first postoperative day relative to the preoperative value. This change arose from the perioperative variations in blood cell counts.

Researchers analyzed the dynamics of the white blood cells in the vascular surgery setting. Chan et al. reported the downregulation of gene expressions of humoral immunity and complement within the neutrophils [26]. Monocytes are vital to the innate immune system and are key in the progression of atherosclerosis from stable to unstable states. A higher circulating monocyte count may signal increased systemic inflammation and ongoing monocyte production [27]. This may suggest a broader inflammatory burden that contributes to vascular injury. Research showed that lymphocytes are crucial for the timely diagnosis of bacteremia and predicting postoperative sepsis [28]. During sepsis onset, apoptosis increased among lymphocytes, leading to a significant drop in B cells and CD4+ and CD8+ T cells [29,30].

In terms of predicting severe AKI, packed red blood cell (RBC) transfusion has shown a good predictive ability in the studied population, with an AUC exceeding 0.9, emerging as a valuable modifiable variable in this setting. Notably, the preoperative hemoglobin concentration did not predict AKI stage 3 in our study. RBC transfusion was associated with an increased risk of developing AKI in many studies [30,31], and this risk appears to be dose-dependent [30]. This association exists both when transfusions are administered alone and when combined with other blood products, as highlighted by Boyko et al. [30]. Moreover, intraoperative erythrocyte transfusions have been identified as independent predictors of AKI following non-cardiac surgery, according to a multicenter, prospective, observational study, even in patients with a low-grade American Society of Anesthesiologists (ASA) physical status (ASA I-II) [32].

Regarding age, Mårtensson et al. have reported that older age was an AKI risk factor in the surgical setting, attributed to decreased renal reserve and an increased prevalence of comorbidities [33]. Surgery duration emerged as an AKI 3 predictor in on-pump aortic surgery in the Dragan et al. study [34]. The same study did not find a significance of preoperative creatinine clearance or patient age in the same setting [34].

In our study, RDW-CV measured early postoperative (AUC 0.750) and on day one after surgery (AUC 0.779) was effective in predicting AKI stage 3. Other research has identified RDW as an AKI predictor in cardiac surgery [35,36], particularly in patients who did not receive transfusions [36]. However, we did not examine this aspect in our cohort. Nonetheless, we emphasize the importance of monitoring dynamic RDW-CV, as it may help predict severe forms of postoperative AKI in major vascular surgery.

The MPV is a key marker of platelet function and activation. Its preoperative (AUC 0.725) and postoperative (AUC 0.759) levels demonstrated strong predictive capability for AKI 3 in our cohort. On day one after surgery, MPV alone did not serve as an AKI3 predictor, but the MPV to P count ratio exhibited predictive value (AUC 0.774). Studies have indicated that MPV can be a cost-effective and more efficient tool than creatinine for the early detection of AKI [37]. MPV at CRRT start was an inexpensive and valuable predictor of 28-day all-cause mortality in patients with AKI needing CRRT [38]. PDW also predicted AKI 3 in our cohort, especially at preoperative (AUC 0.755), early postoperative setting (AUC 0.736), and to a lesser extent in day one after surgery (AUC < 0.7). Platelet activation leads to morphological changes, and PDW can indicate the presence of microthrombi in the microcirculation, offering insights into tissue perfusion and reinforcing its role as an AKI predictor [39].

Dynamic monitoring of inflammatory indices is crucial, as our research highlighted DeltaNLR_0_preop as a predictor for AKI 3, not just a single NLR value. High NLR was previously linked to a greater risk of AKI 3 and the need for CRRT in septic patients [40] and those undergoing cardiac surgery [41].

Regarding the preoperative risk scores, we found that VSG-CRI (AUC 0.859, *p* = 0.001) outperformed RCRI (AUC 0.741, *p* = 0.038) in predicting AKI 3. While the RCRI was primarily designed to assess the risk of cardiovascular complications [12], it has also demonstrated effectiveness in predicting other postoperative complications [42]. Previous studies have shown that the VSG-CRI more accurately predicts both cardiac complications and in-hospital mortality than the RCRI in the context of vascular surgery [11,43].

Our study has several limitations. This is a single-center retrospective study. Since we have not predetermined the sample size, we chose the consecutive patients approach to minimize potential selection bias. Although the missing data could introduce additional biases, its overall impact on the study was negligible, as only two patients were excluded due to missing information. All patients in our analyzed cohort underwent aorto-bifemoral bypass; however, popliteal extension was performed for those with significant multilevel occlusive disease. This detail affects the homogeneity of the cohort. In our analysis, the AKI assessment was based solely on creatinine measurements, without considering urine output. It is essential to note the exceedingly low absolute count of AKI Stage 3 cases, which notably significantly undermines the robustness of our predictive analysis. Additionally, there were institutional limitations that affected our analysis, including inconsistencies in data related to various inflammatory indicators, such as levels of protein C and fibrinogen. We also lacked detailed information on transfusion requirements (including the type of product, timing, and triggers), aortic cross-clamping duration, as well as hemodynamic and volemic status data, including the use of vasopressors. We only used RDW-CV in our analysis, as RDW-SD data was inconsistently available. Additionally, we cannot provide information about the timing of AKI occurrence in the postoperative course, and its duration. These limitations hindered our ability to conduct a more comprehensive analysis. Moreover, our study lacks external validation of the findings, which should be carefully considered when interpreting the results. To validate our findings, it is essential to conduct future prospective multicenter studies.

Future research may identify cost-effective blood tests for assessing AKI risk in patients undergoing major vascular surgery. Studies should examine the links between serum cardiac and inflammatory biomarkers, fibrinogen levels, nutritional status, preoperative medications, and surgical techniques, all in relation to postoperative AKI. An important area of research could focus on therapeutic interventions. A promising area of research lies in tailoring therapeutic interventions; for instance, glutamine has shown potential in preventing postoperative AKI [44]. The glutamine levels dropped significantly post-vascular surgery but typically recovered within five days [45], with lower glutamine levels associated with worsened atherosclerotic lesions [46].

We utilized a dynamic approach to assess postoperative AKI in patients undergoing aorto-bifemoral bypass surgery for Leriche syndrome, incorporating routine blood analyses and clinical patient characteristics. Our findings could serve as a valuable, cost-effective tool for clinicians managing postoperative AKI, enabling personalized care for complex patients, particularly those undergoing major vascular surgery. However, it is important to emphasize that our results necessitate future prospective research and external validation.

## 5. Conclusions

We highlight the potential value of this dynamic and cost-effective approach to integrating specific routine perioperative variables for AKI risk assessment in clinical practice. The SIRI change between the day after surgery and preoperative measurements, along with age and preoperative creatinine clearance, were identified as independent factors for postoperative AKI in patients undergoing aorto-bifemoral bypass for aorto-iliac occlusive disease. The number of packed red blood cells transfused best predicted the development of AKI stage 3, followed by various routine perioperative factors. However, further prospective confirmation and external validation of these findings are necessary.

## Figures and Tables

**Table 1:** Patient characteristics (only the variable with significantly different values in the two subgroups and the clinically relevant characteristics).

Variable	No_AKI Patients (73 Patients)	AKI Patients (41 Patients)	*p* ^1^
Age (years) ^2^	59 [55–63]	64 [58.5–68]	0.001
Male sex ^3^	66 (90.41%)	37 (90.24%)	1
Leriche–Fontaine IV ^3^	31 (42.46%)	13 (31.70%)	0.318
G&P anesthesia ^3^	45 (61.64%)	23 (56.09%)	0.691
Popliteal bypass extension ^3^	21 (28.76%)	13 (31.70%)	0.833
Hb_preop ^2^	12.7 [11.8–13.2]	12.9 [12.3–13.4]	0.100
Surgical reintervention ^3^	10 (13.70%)	9 (21.95%)	0.434
Creat_clearance_preop (mL/min) ^2^	101 [95.5–106]	87 [63–102]	0.001
RCRI ^2^	2 [1,2]	2 [1,2]	0.004
VSG-CRI ^2^	4 [2,3,4,5]	5 [4,5,6,7]	0.006
Intraop_time (h) ^2^	4 [3,4,5,6]	6 [4,5,6,7,8]	0.001
NLR_1 ^2^	7.54 [5.00–10.21]	10.35 [6.14–15.39]	0.018
MLR_1 ^2^	0.68 [0.50–0.93]	0.89 [0.61–1.20]	0.038
PDW_preop (fl) ^2^	12 [10.40–13.15]	13.10 [11.60–14.75]	0.007
MPV_preop (fl) ^4^	10.18 (±1.16)	10.70 (±1.02)	0.008
PDW_0 (fl) ^2^	10.90 [9.65–12.50]	12.60 [11.20–13.30]	0.002
MPV_0 (fl) ^2^	10 [9.30–10.70]	10.80 [9.80–11.10]	0.001
PDW_1 (fl) ^2^	11.70 [10.30–13.30]	13.30 [11.70–14.65]	0.007
MPV_1 (fl) ^2^	10.30 [9.75–11.20]	11.10 [10.35–11.70]	0.005
P_0 (×10^3^/µL)	207 [173.5–259.5]	180 [148–204.5]	0.023
P_1 (×10^3^/µL) ^2^	209 [169.5–261]	182 [149.5–223]	0.049
MPV/P_preop ^2^	0.03 [0.02–0.04]	0.04 [0.03–0.05]	0.024
MPV/P_0 ^2^	0.04 [0.03–0.06]	0.05 [0.04–0.07]	0.004
MPV/P_1 ^2^	0.05 [0.03–0.06]	0.06 [0.04–0.07]	0.019
MPV/Lf_1 ^2^	9.67 [6.64–14.10]	13.79 [8.48–20]	0.023
DeltaMLR_1_preop ^2^	0.34 [0.20–0.55]	0.53 [0.28–0.76]	0.037

^1^ *p* value Mann–Whitney test or exact Fisher test; ^2^ median [IQR]; ^3^ number (%); ^4^ mean (± standard deviation. Note: “×” is a multiplication sign; _preop, preoperative value; _0, value measured upon intensive care admission; and _1, value recorded on the day after surgery. Abbreviations: AKI, acute kidney injury; Creat_clearance_preop, preoperative creatinine clearance; DeltaMLR_1_preop, day one after surgery–preoperative MLR change; G&P anesthesia, general and peridural anesthesia; Hb_preop, preoperative hemoglobin; Intraop_time, surgery duration; MLR, monocyte-to-lymphocyte ratio; MPV, mean platelet volume; NLR, neutrophil-to-lymphocyte ratio; P, platelet count; PDW, platelet distribution width; RCRI, Revised Cardiac Risk Index; VSG-CRI, Vascular Study Group Cardiac Risk Index.

**Table 2:** The binary logistic regression results (endpoint AKI occurrence).

Variable	Univariable Analysis		Multivariable Analysis	
	*p*	OR (CI95%)	*p*	OR (CI95%)
NLR_1 ^1^	0.021	1.091 (1.013–1.176)		
PDW_preop ^1^	0.012	1.254 (1.050–1.498)	0.420	
MPV_preop ^1^	0.008	1.665 (1.144–2.424)	0.927	
PDW_0 ^1^	0.009	1.285 (1.066–1.550)		
MPV_0 ^1^	0.003	1.891 (1.246–2.871)		
PDW_1 ^1^	0.016	1.224 (1.038–1.444)		
Age ^1^	0.002	1.106 (1.036–1.181)	0.575	
Creat_clearance_preop ^2^	0.001	1.046 (1.021–1.070)	0.009	1.037 (1.009–1.066)
RCRI ^1^	0.002	1.910 (1.261–2.894)	0.671	
VSG-CRI ^1^	0.005	1.333 (1.092–1.627)	0.940	
PRBCs ^1^	0.023	1.520 (1.060–2.182)	0.682	
Intraop_time ^1^	0.001	1.479 (1.188–1.843)	0.008	1.435 (1.100–1.873)
DeltaNLR_1_preop ^1^	0.033	1.092 [1.007–1.184]		
DeltaSIRI_1_preop ^1^	0.041	1.070 (1.003–1.142)	0.021	1.080 (1.012–1.152)

^1^ The variable presents a direct relationship with the endpoint. ^2^ The variable presents an inverse relationship with the endpoint. Note: _preop refers to preoperative variable; _0 refers to variable upon ICU admission; and _1 refers to day-one-after-surgery variable. Abbreviations: Creat_clearance_preop, preoperative creatinine clearance; DeltaNLR_1_preop, day-one-after-surgery–preoperative NLR change; DeltaSIRI_1_preop, day-one-after-surgery–preoperative systemic inflammatory response index change; Intraop_time, intraoperative duration; MPV, mean platelet volume; NLR, neutrophil-to-lymphocyte ratio; PDW, platelet distribution width; PRBCs, packed red blood cells; RCRI, Revised Cardiac Risk Index; VSG-CRI, Vascular Study Group Cardiac Risk Index.

**Table 3:** Significant results of the tested variables in ROC analysis (endpoint AKI3).

Variable	AUC	*p*	CI 95%
PRBCs ^1^	0.924	0.001	0.856–0.992
Age ^1^	0.895	0.001	0.786–0.899
Intraop_time ^1^	0.895	0.001	0.781–0.899
VSG-CRI ^1^	0.859	0.001	0.716–0.899
RDW-CV_1 ^1^	0.779	0.044	0.507–0.980
MPV/P_1 ^1^	0.774	0.001	0.625–0.923
N_0 ^1^	0.774	0.001	0.633–0.915
Creat_clearance_preop ^2^	0.763	0.002	0.594–0.931
MPV_0 ^1^	0.759	0.001	0.621–0.897
PDW_preop	0.755	0.001	0.610–0.899
RDW-CV_0 ^1^	0.750	0.035	0.518–0.982
RCRI ^1^	0.741	0.038	0.513–0.969
PDW_0 ^1^	0.736	0.003	0.582–0.890
MPV_preop ^1^	0.725	0.015	0.544–0.906
L_0 ^1^	0.725	0.026	0.526–0.924
DeltaNLR_0_preop ^1^	0.707	0.031	0.519–0.894
NLR_0 ^1^	0.695	0.044	0.505–0.886
PDW_1 ^1^	0.694	0.035	0.514–0.874
DeltaSII_0_preop ^1^	0.655	0.011	0.536–0.773

^1^ We report the ROC analysis to endpoint AKI 3 occurrence. ^2^ We report the ROC analysis to endpoint AK 3 absent (inverse relationship between the variables). Note: _preop refers to preoperative variable; _0 refers to variable upon ICU admission; and _1 refers to day-one-after-surgery variable Abbreviations: AKI, acute kidney injury; AUC, area under the curve; DeltaNLR_0_preop, early postoperative–preoperative NLR change; DeltaSII_0_preop, early postoperative–preoperative SII change; Intraop_time, intraoperative duration; L, leukocytes; MPV, mean platelet volume; N, neutrophils count; NLR, neutrophil-to-lymphocyte ratio; PDW, platelet distribution width; PRBCs, packed red blood cells; RCRI, Revised Cardiac Risk Index; RDW-CV, red cell distribution width coefficient of variation; VSG-CRI, Vascular Study Group Cardiac Risk Index.

## Data Availability

The data presented in this study are available on request from the corresponding author, due to privacy and ethical restrictions.

## Supplementary Materials

The following supporting information can be downloaded at: https://www.mdpi.com/article/10.3390/diagnostics16091382/s1, Table S1: Patients data recorded in patients with and without postoperative AKI; Table S2: The logistic binary regression analysis (endpoint AKI occurrence); Table S3: The results of ROC analysis regarding AKI 3 prediction.
