# Supplementary material for: A Dynamic Approach to Assessing and Predicting AKI Risk in Patients with Aortoiliac Occlusive Disease Undergoing Aorto-Bifemoral Bypass
Source: Diagnostics (Basel). 2026 May 1;16(9):1382. doi: 10.3390/diagnostics16091382 (PMC13163281; doi:10.3390/diagnostics16091382)
Supplement: Supplementary file 1 [file diagnostics-16-01382-s001.zip › diagnostics-4250775-supplementary.pdf]

Table S1. Patients data recorded in patients with and without postoperative AKI

Variable	No_AKI patients (73 patients)	AKI patients (41 patients)	*P* [1]
Age (years) [2]	59 [55-63]	64 [58.5-68]	0.001
Male sex [3]	66 (90.41%)	37(90.24%)	1
Leriche-Fontaine IV [3]	31 (42.46%)	13 (31.70%)	0.318
Creat_clearance_preop (ml/min) [2]	101 [95.5-106]	87 [63-102]	0.001
RCRI [2]	2 [1-2]	2 [1-2]	0.004
VSG-CRI [2]	4 [2-5]	5 [4-7]	0.006
Intraop_time (hours) [2]	4 [3-6]	6 [4-8]	0.001
G&P anesthesia [3]	45 (61.64%)	23 (56.09%)	0.691
Popliteal bypass extension	21 (28.76%)	13 (31.70%)	0.833
PRBCs (units) [2]	0 [0-1]	1 [0-2]	0.061
Surgical reintervention [3]	10 (13.70%)	9 (21.95%)	0.434
CK_preop (U/l) [2]	76 [58-114.50]	73 [52.50 -119]	0.643
CK_0 (U/l) [2]	77 [63-147]	77 [57-113.50]	0.524
CK_1 (U/l) [2]	475 [235.5-1241.50]	761 [215-2140.50]	0.277
SII_preop [2]	608.34 [423.35-941.21]	628.24 [444.63-854.46]	0.918
SIRI_preop [2]	1.75 [1.16-2.45]	1.75 [1.21-2.76]	0.448
AISI_preop [2]	475.46 [306.06- 721.79]	433.39 [305.73-788.27]	0.811
NLR_preop [2]	2.33 [1.56-2.88]	2.36 [1.72-3.02]	0.466
MLR_preop [2]	0.30 [0.24-0.40]	0.31 [0.24-0.45]	0.549
PLR_preop [2]	113.44 [84.77-154.37]	105.55 [86.22 -148.65]	0.626
SII_0 [2]	1091.29 [657.01-1728.38	1005.94 [745.01-1640.46]	0.936
SIRI_0 [2]	2.57 [1.54-7.48]	4.24 [2.36-7.30]	0.134
AISI_0 [2]	573.46 [310.76-1632.57]	694.09 [373.27-1398.61]	0.553
NLR_0 [2]	4.88 [3.72-7.63]	5.60 [4.19-8.51]	0.120
MLR_0 [2]	0.35 [0.22-0.61]	0.46 [0.29-0.62]	0.197
PLR_0 [2]	126.05 [88.09-188.92]	120.83 [77.96-158.40]	0.292
SII_1 [2]	1530.76 [937.32-2397.31]	1702.14 [1018.58-2824.31]	0.361
SIRI_1 [2]	5.35 [3.52-7.85]	6.38 [4.25-9.90]	0.131
AISI_1 [2]	1125.10 [678.87-1916.44]	1058.20 [687.32-1973.51]	0.890
NLR_1 [2]	7.54 [5.00-10.21]	10.35 [6.14-15.39]	0.018
MLR_1 [2]	0.68 [0.50-0.93]	0.89 [0.61-1.20]	0.038
PLR_1 [2]	189.78 [121.30-307.70]	202.43 [153.09-297.05]	0.557
Hb_preop [2]	12.7 [11.8-13.2]	12.9 [12.3-13.4]	0.100
RDW-CV_preop (%) [2]	13.30 [12.60-14.2]	13.50 [12.80-14.40]	0.476
PDW_preop (fl) [2]	12 [10.40-13.15]	13.10 [11.60-14.75]	0.007
MPV_preop (fl) [4]	10.18(±1.16)	10.70 (±1.02)	0.008
RDW-CV_0 (%)[2]	13.30 [12.60-13.45]	13.60 [12.80-14.55]	0.401
PDW_0 (fl) [2]	10.90 [9.65-12.50]	12.60 [11.20-13.30]	0.002

MPV_0 (fl) [2]	10 [9.30-10.70]	10.80 [9.80-11.10]	0.001
RDW-CV_1 (%)[2]	13.30 [12.70-14.60]	13.80[13.05-15.30]	0.267
PDW_1 (fl) [2]	11.70 [10.30-13.30]	13.30 [11.70-14.65]	0.007
MPV_1 (fl) [2]	10.30 [9.75-11.20]	11.10 [10.35 -11.70]	0.005
L_preop ($x10^3$/µl) [2]	8.97 [7.76-10.92]	9.44 [7.83-10.57]	0.904
N_preop ($x10^3$/µl) [2]	5.33 [4.48-6.82]	5.88 [4.39-6.99]	0.424
M_preop ($x10^3$/µl) [2]	0.77 [0.60-0.98]	0.77 [0.65-0.92]	0.730
Lf_preop ($x10^3$/µl) [2]	2.44 [1.09-2.95]	2.35 [1.92-3.11]	0.628
P_preop ($x10^3$/µl) [2]	293 [220.5-358.50]	241 [208.5-313.5]	0.093
L_0 ($x10^3$/µl) [2]	10.62 [8.33-13.95]	11.70 [9.19-15.98]	0.226
N_0 ($x10^3$/µl) [2]	8.49 [6.40-11.40]	9.45 [7.26-12.36]	0.114
M_0 ($x10^3$/µl) [2]	0.57 [0.34-1]	0.69 [0.45-1.06]	0.275
Lf_0 ($x10^3$/µl) [2]	1.63 [1.15-2.41]	9.45 [7.26-12.36]	0.569
P_0 ($x10^3$/µl) [2]	207 [173.5-259.5]	180 [148-204.5]	0.023
L_1 ($x10^3$/µl) [2]	10.13 [8.31-12.12]	10.28 [8.25-12.38]	0.939
N_1 ($x10^3$/µl) [2]	7.88 [6.63-9.65]	8.44 [6.46-9.99]	0.690
M_1 ($x10^3$/µl) [2]	0.75 [0.56-0.96]	0.67 [0.51-0.96]	0.601
Lf_1 ($x10^3$/µl) [2]	1.13 [0.69-1.53]	0.87 [0.58-1.34]	0.057
P_1 ($x10^3$/µl) [2]	209 [169.5-261]	182 [149.5-223]	0.049
MPV/P_preop [2]	0.03 [0.02-0.04]	0.04 [0.03-0.05]	0.024
MPV/P_0 [2]	0.04 [0.03-0.06]	0.05 [0.04-0.07]	0.004
MPV/P_1 [2]	0.05 [0.03-0.06]	0.06 [0.04-0.07]	0.019
MPV/Lf_preop [2]	4.07 [3.30-5.36]	4.76 [3.35-5.73]	0.214
MPV/Lf_0 [2]	6.15 [4.14-8.97]	7.20 [4.73-9.95]	0.225
MPV/Lf_1 [2]	9.67 [6.64-14.10]	13.79 [8.48-20]	0.023
DeltaSII_0_preop [2]	454.98 [50.97-1004.90]	449 [270.50-636.80]	0.904
DeltaSII_1_preop [2]	915.51 [296-1737.71]	1116.57 [411.33-2112.88]	0.284
DeltaSIRI_0_preop [2]	1.22 [-0.09 – 4.91]	1.95 [0.66-4.19]	0.510
DeltaSIRI_1_preop [2]	3.33 [1.71-5.62]	5.11 [2.26-7.88]	0.131
DeltaAISI_1_preop [2]	532.04 [201.34-1212.63]	701.73 [195.21-1351.91]	0.473
DeltaAISI_0_preop [2]	101.20 [-172.91 – 1025.45]	234.07 [-88.03 -719.94]	0.510
DeltaPLR_0_preop [2]	7.25 [-24.03-68.68]	2.52 [-11.77-31.50]	0.806
DeltaPLR_1_preop [2]	83.90 [26.89-162.71]	86.28 [41.84-168.13]	0.403
DeltaMLR_0_preop [2]	0.05 [-0.07-0.26]	0.07 [0-0.28]	0.328
DeltaMLR_1_preop [2]	0.34 [0.20-0.55]	0.53 [0.28-0.76]	0.037
DeltaNLR_0_preop [2]	2.77 [1.17-5.05]	3.59 [2.15-5.52]	0.147
DeltaNLR_1_preop [2]	5.14 [2.68-7.50]	7.63 [3.88-12.68]	0.021

[1] *p* value Mann–Whitney test or exact Fisher test; [2] median [IQR]; [3] number (%); [4] mean (± standard deviation). Note: "x" is a multiplication sign; _preop, preoperative value; _0, value measured upon intensive care admission; _1, value recorded in the day one after surgery; Delta, variable change in the two perioperative moments. Abbreviations: AISI, aggregate index of systemic inflammation; AKI, acute kidney injury; CK, creatine kinase; creat_clearance_preop, preoperative creatinine clearance; G&P anesthesia, general and peridural anesthesia; Hb_preop, preoperative hemoglobine; Intraop_time, surgery duration; L, leukocytes count; Lf, lymphocytes; M, monocytes count; MLR, monocyte-to-lymphocyte ratio; MPV, mean platelet volume; N, neutrophils count; NLR, neutrophil-to-lymphocyte ratio; P, platelets count; PDW, platelet distribution width; PLR, platelets-to-lymphocyte ratio; PRBCs, packed red blood cells; RCRI, Revised Cardiac Risk Index ; RDW-CV, red cell distribution width coefficient of variation; SII, systemic inflammatory index; SIRI, systemic inflammatory response index VSG-CRI, Vascular Surgery Cardiac Risk Index.

Table S2. The logistic binary regression analysis (endpoint AKI occurence)

Variable	Univariable analysis		Multivariable analysis	
	p	OR (CI95%)	p	OR (CI95%)
Age (years) [1]	0.002	1.106 (1.036-1.181)	0.575	0.724
Male sex [1]	0.977			
Leriche-Fontaine IV [1]	0.259			
Creat_clearance_preop[2]	0.001	1.046 (1.021-1.070)	0.009	1.037 (1.009-1.066)
RCRI [1]	0.002	1.910 (1.261-2.894)	0.671	
VSG-CRI [1]	0.005	1.333 (1.092-1.627)	0.940	
Intraop_time (hours) [1]	0.001	1.479 (1.188-1.843)	0.008	1.435 (1.100-1.873)
G&P anesthesia [1]	0.563			
Bypass extension[1]	0.777			
PRBCs (units) [1]	0.023	1.520 (1.060-2.182)	0.682	
Surgical reintervention [1]	0.316			
CK_preop[1]	0.486			
CK_0[1]	0.679			
CK_1[1]	0.092			
SII_preop[1]	0.623			
AISI_preop[1]	0.760			
NLR_preop[1]	0.215			
MLR_preop[1]	0.227			
PLR_preop[1]	0.249			
SII_0[1]	0.494			
SIRI_0[1]	0.282			
AISI_0[1]	0.939			
NLR_0[1]	0.512			
MLR_0[1]	0.349			
PLR_0[2]	0.175			
SII_1[1]	0.943			
SIRI_1[1]	0.068			
AISI_1[1]	0.512			
NLR_1[1]	0.021	1.091(1.013-1.176)		
MLR_1[1]	0.070			
PLR_1[2]	0.593			
RDW_preop[1]	0.439			
PDW_preop[1]	0.012	1.254 (1.050-1.498)	0420	
MPV_preop[1]	0.008	1.665 (1.144-2.424)	0927	
RDW_0[1]	0.702			
PDW_0[1]	0.009	1.285 (1.066-1.550)		
MPV_0[1]	0.003	1.891 (1.246-2.871)		

RDW_1[1]	0.472			
PDW_1[1]	0.016	1.224 (1.038-1.444)		
MPV_1[1]	0.141			
Hb_preop[1]	0.123			
L_preop[1]	0.520			
N_preop[1]	0.354			
M_preop[1]	0.443			
Lf_preop[1]	0.664			
P_preop[1]	0.141			
L_0[1]	0.412			
N_0[1]	0.287			
M_0[1]	0.313			
Lf_0[1]	0.433			
P_0[2]	0.054			
L_1[1]	0.196			
N_1[1]	0.185			
M_1[1]	0.404			
Lf_1[2]	0.062			
P_1[2]	0.056			
MPV/P_preop[1]	0.360			
MPV/P_0[1]	0.074			
MPV/P_1[1]	0.060			
MPV/Lf_preop[1]	0.125			
MPV/Lf_0[1]	0.624			
MPV/Lf_1[1]	0.250			
DeltaNLR_0_preop[1]	0.820			
DeltaNLR_1_preop[1]	0.033	1.092 [1.007-1.184]		
DeltaSII_0_preop[1]	0.464			
DeltaSII_1_preop[1]	0.779			
DeltaSIRI_0_preop[1]	0.634			
DeltaSIRI_1_preop[1]	0.041	1.070 (1.003-1.142)	0.021	1.080 (1.012-1.152)
DeltaAISI_1_preop[1]	0.397			
DeltaAISI_0_preop[1]	0.628			
DeltaPLR_0_preop[2]	0.402			
DeltaPLR_1_preop[1]	0.954			
DeltaMLR_0_preop[1]	0727			
DeltaMLR_1_preop[1]	0.117			

[1] The variable presents a direct relationship with the endpoint.; [2]The variable presents an inverse relationship with the endpoint. Note: _preop, preoperative value; _0, value measured upon intensive care admission; _1, value recorded in the day one after surgery; Delta, variable change in the two perioperative moments. Abbreviations: AISI, aggregate index of systemic inflammation; AKI, acute kidney injury; CK, creatine kinase; creat_clearance_preop, preoperative creatinine clearance; G&P anesthesia, general and peridural anesthesia; Intraop_time, surgery duration; L, leukocytes count; Lf, lymphocytes; M, monocytes count; MLR, monocyte-to-lymphocyte ratio; MPV, mean platelet volume; N, neutrophils count; NLR, neutrophil-to-lymphocyte ratio; P, platelets count; PDW, platelet distribution width; PLR, platelets-to-lymphocyte ratio; PRBCs, packed red blood cells; RCRI, Revised Cardiac Risk Index ; RDW-CV, red cell distribution width coefficient of variation; SII, systemic inflammatory index; SIRI, systemic inflammatory response index VSG-CRI, Vascular Surgery Cardiac Risk Index.

Table S3. The results of ROC analysis regarding AKI 3 prediction

	AUC	*p*	CI 95%
Age [1]	0.895	0.001	0.786-0.899
Creat_clearance_preop [2]	0.763	0.002	0.594-0.931
RCRI [1]	0.741	0.038	0.513-0.969
VSG-CRI [1]	0.859	0.001	0.716-0.899
PRBCs [1]	0.924	0.001	0.856-0.992
Intraop_time [1]	0.895	0.001	0.781-0.899
CK_preop [1]	0.511	0.952	
CK_0 [1]	0.519	0.917	
CK_1 [1]	0.430	0.669	
SII_preop [1]	0.514	0.891	
SIRI_preop [1]	0.591	0.313	
AISI_preop [1]	0.555	0.650	
NLR_preop [1]	0.548	0.502	
MLR_preop [1]	0.514	0.920	
PLR_preop [1]	0.430	0.496	
SII_0 [1]	0.614	0.147	
SIRI_0 [1]	0.643	0.066	
AISI_0 [1]	0.573	0.556	
NLR_0 [1]	0.695	0.044	0.505-0.886
MLR_0 [1]	0.541	0.665	
PLR_0 [2]	0.420	0.464	
SII_1 [1]	0.459	0.771	
SIRI_1 [1]	0.568	0.679	
AISI_1 [1]	0.491	0.951	
NLR_1 [1]	0.520	0.901	
MLR_1 [1]	0.473	0.843	
PLR_1 [2]	0.352	0.215	
Hb_preop [1]	0.589	0.315	
RDW_preop [1]	0.575	0.553	
PDW_preop [1]	0.755	0.001	0.610-0.899
MPV_preop [1]	0.725	0.015	0.544-0.906
RDW_0 [1]	0.750	0.035	0.518-0.982
PDW_0 [1]	0.736	0.003	0.582-0.890
MPV_0 [1]	0.759	0.001	0.621-0.897
RDW_1 [1]	0.779	0.044	0.507-0.980
PDW_1 [1]	0.694	0.035	0.514-0.874
MPV_1 [1]	0.654	0.110	
L_preop [1]	0.544	0.733	
N_preop [1]	0.588	0.466	
M_preop [1]	0.594	0.241	
Lf_preop [1]	0.528	0.832	
P_preop [1]	0.465	0.770	
L_0 [1]	0.725	0.026	0.526-0.924

N_0 [1]	0.774	0.001	0.633-0.915
M_0 [1]	0.566	0.654	
Lf_ [1]	0.502	0.987	
P_0 [2]	0.427	0.564	
L_1 [1]	0.534	0.824	
N_1 [1]	0.580	0.632	
M_1 [1]	0.433	0.686	
Lf_1 [1]	0.491	0.936	
P_1 [2]	0.270	0.051	
MPV/P_preop [1]	0.603	0.214	
MPV/P_0 [1]	0.635	0.196	
MPV/P_1 [1]	0.774	0.001	0.625-0.923
MPV/Lf_preop [1]	0.532	0.806	
MPV/Lf_0 [1]	0.544	0.756	
MPV/Lf_1 [1]	0.528	0.773	
DeltaNLR_0_preop [1]	0.707	0.031	0.519-0.894
DeltaNLR_1_preop [1]	0.520	0.905	
DeltaSII_0_preop [1]	0.655	0.011	0.536-0.773
DeltaSII_1_preop [1]	0.655	0.609	
DeltaSIRI_0_preop [1]	0.648	0.085	
DeltaSIRI_1_preop [1]	0.559	0.720	
DeltaAISI_1_preop [1]	0.475	0.855	
DeltaAISI_0_preop [1]	0.591	0.420	
DeltaPLR_0_preop [1]	0.486	0.821	
DeltaPLR_1_preop [1]	0.361	0.310	
DeltaMLR_0_preop [1]	0.500	0.900	
DeltaMLR_1_preop [1]	0.432	0.647	

[1] We report the ROC analysis to endpoint AKI 3 occurrence. [2] We report the ROC analysis to endpoint AKI 3 absent (inverse relationship between the variables). Note: _preop, preoperative value; _0, value measured upon intensive care admission; _1, value recorded in the day one after surgery; Delta, variable change in the two perioperative moments. Abbreviations: AISI, aggregate index of systemic inflammation; AKI, acute kidney injury; CK, creatine kinase; creat_clearance_preop, preoperative creatinine clearance; G&P anesthesia, general and peridural anesthesia; Hb_preop, preoperative hemoglobine; Intraop_time, surgery duration; L, leukocytes count; Lf, lymphocytes; M, monocytes count; MLR, monocyte-to-lymphocyte ratio; MPV, mean platelet volume; N, neutrophils count; NLR, neutrophil-to-lymphocyte ratio; P, platelets count; PDW, platelet distribution width; PLR, platelets-to-lymphocyte ratio; PRBCs, packed red blood cells; RCRI, Revised Cardiac Risk Index ; RDW-CV, red cell distribution width coefficient of variation; SII, systemic inflammatory index; SIRI, systemic inflammatory response index VSG-CRI, Vascular Surgery Cardiac Risk Index.
